# Spin slush in an extended spin ice model

**DOI:** 10.1038/ncomms12234

**Published:** 2016-07-29

**Authors:** Jeffrey G. Rau, Michel J. P. Gingras

**Affiliations:** 1 Department of Physics and Astronomy, University of Waterloo, Waterloo, Ontario, Canada N2L 3G1; 2 Perimeter Institute for Theoretical Physics, Waterloo, Ontario, Canada N2L 2Y5; 3 Canadian Institute for Advanced Research, 180 Dundas Street West, Suite 1400, Toronto, Ontario, Canada M5G 1Z8

## Abstract

We present a new classical spin liquid on the pyrochlore lattice by extending spin ice with further neighbour interactions. We find that this disorder-free spin model exhibits a form of dynamical heterogeneity with extremely slow relaxation for some spins, while others fluctuate quickly down to zero temperature. We thus call this state spin slush, in analogy to the heterogeneous mixture of solid and liquid water. This behaviour is driven by the structure of the ground-state manifold which extends the celebrated two-in/two-out ice states to include branching structures built from three-in/one-out, three-out/one-in and all-in/all-out tetrahedra defects. Distinctive liquid-like patterns in the magnetic correlations serve as a signature of this intermediate range order. Possible applications to materials as well the effects of quantum tunnelling are discussed.

## Abstract (web summary)

The ultimate understanding of glassy phenomena will impact fields ranging from materials science to biophysics. Here, the authors report a heterogeneous mixture of slow relaxing spins and fast fluctuating spins, in analogy to slush as a mixture of ice and liquid water.

## Graphical abstract

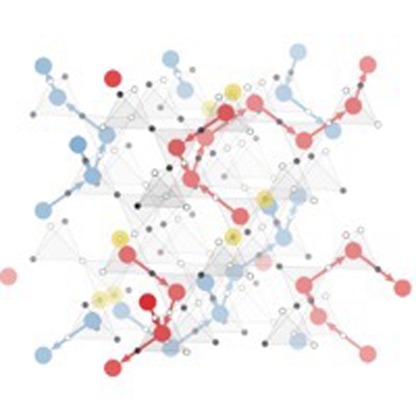

The physics of glasses plays an important role in many types of physical systems; from its origins in the physics of liquids^1^ further realizations have been found in disordered magnets^2^, superconductors^3^ and metals^4^ through to soft-condensed matter^5^ and even biophysics^6^. While ubiquitous, a complete understanding of glasses remains an important open problem in condensed matter physics. Connections between these vastly different contexts have proven fruitful in making progress. For example, studying conceptually and computationally simpler spin models, may inform the physics of super-cooled liquids and structural glasses^1^. However, there are complications—while spin glasses are driven by the combination of random quenched disorder and frustration^2^, glass-forming liquids are intrinsically disorder-free^1^. Finding a disorder-free spin model that realizes the diverse range of phenomena observed in glass formers, such as the dramatic slowing down of relaxation and emergence of spatially heterogeneous dynamics, is a serious challenge. Some examples of disorder-free spin models with strong freezing have been proposed^7^,^8^,^9^,^10^,^11^,^12^. Each of these proposals has some limitations, be it the lack of heterogeneous dynamics, the need for multi-spin interactions, the use of uncontrolled approximations or the introduction of non-local dynamics.

In this article, we introduce a cooperative paramagnet that we call spin slush (SS) which appears in an extended spin ice (ESI) model. This classical SS model is disorder-free and includes only first-, second- and third-neighbour Ising bilinear exchange interactions and thus lacks the shortcomings discussed above. We start from spin ice (SI), a well-studied magnetic analogue of common water ice^13^, magnetic moments pointing in or out of the corner-shared tetrahedra of the pyrochlore lattice embody the proton displacements of water ice^14^. Similar to water ice, SI displays an extensive ground-state degeneracy, and thus an associated extensive residual entropy, characterized by the two-in/two-out ice rule condition on each tetrahedron^13^. In SS, we find that the ground-state manifold of SS is larger than that of SI and contains a far richer set of states. In addition to the two-in/two-out tetrahedra of the SI ground-state manifold, there are spatially extended structures assembled from three-in/one-out, three-out/one-in and all-in/all-out tetrahedra. Built from SI defects, these structures are not simply loops or strings, but include branching tree-like objects. After characterizing the static thermodynamic and magnetic properties of SS, we turn to dynamics. Approaching zero temperature, we find freezing, as in SI^15^,^16^, with an exponentially increasing average relaxation time. However, unlike in SI where all of the spins freeze uniformly as the temperature is lowered, the spins in the SS exhibit highly heterogeneous dynamics reminiscent of glass formers^17^. While many of the spins strongly freeze with an extremely slow relaxation rate, a fraction of the spins, organized into spatially local clusters, remain completely dynamic, relaxing almost immediately. Since this model is disorder-free, the random distribution of these dynamical spins derives solely from the overall freezing behaviour. This dynamical heterogeneity in SS at low temperatures motivates the name SS, in analogy to slush where liquid water and solid ice coexist as a mixture. Finally, we speculate on the behaviour of quantum SS as well as possible experimental relevance in frustrated pyrochlore magnets.

## Results

### Model

We start with a review of some key results for the nearest-neighbour SI model^18^ to establish our notation and motivate the SS model. The SI model is a nearest-neighbour Ising antiferromagnet on the pyrochlore lattice, with Hamiltonian 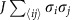, where *σ*_*i*_=±1 are the Ising spins. This can be reformulated in terms of ice rule defects, or charges, defined on each tetrahedron. With each tetrahedron identified with a dual diamond lattice site *I*, one defines the charge 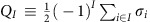, where (−1)^*I*^ is a sign reflecting the sublattice of *I*. In this language, the nearest-neighbour SI Hamiltonian simply penalizes non-zero charges, taking the form


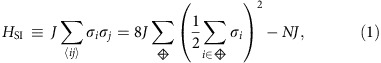



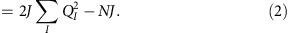


The ground states of this model are those with *Q*_*I*_=0 for all tetrahedra, i.e., the celebrated two-in/two-out states of the ice manifold. This manifold is macroscopically degenerate with a residual entropy given approximately by 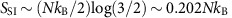 (ref. 13). Because of this extensive ground-state degeneracy, addition of small perturbations will generically select an ordered state from this manifold at low temperatures^13^.

To explore the effects of such perturbations, we consider the addition of second- and third-neighbour Ising exchanges of the form


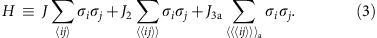


**Figure 1:**
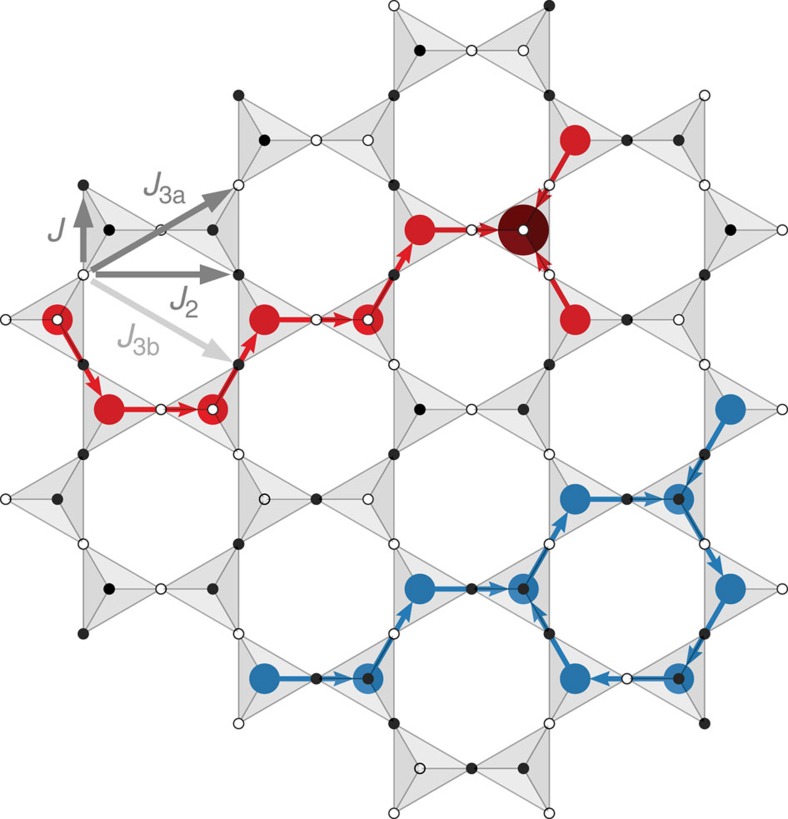
Example of spin slush ground state. A spin slush ground state that includes all instances of the rules discussed in the main text. The first (*J*), second (*J*_2_) and third (*J*_3a_, *J*_3b_) neighbour exchange paths are indicated. The colours indicate *σ*_*i*_=±1 (black, white) for the pyrochlore sites, and the charge *Q*_*I*_ for the dual lattice with *Q*_*I*_=0 (grey), *Q*_*I*_=±1 (red, blue) and *Q*_*I*_=±2 (dark red, dark blue). The arrow passes through the location of the minority spin for a single charge. This state contains branching lines of charge of both signs, a charge loop and a double-charge tetrahedron.

For third-neighbour exchange, we include only those that are composed of two nearest-neighbour steps, as illustrated in Fig. 1 and Supplementary Fig. 1. For many mechanisms that generate such interactions in real materials, for example super-exchange or through virtual crystal field excitations, one expects the interactions *J*_2_ and *J*_3a_ to be generated on equal footing. The other third-neighbour exchange, *J*_3b_, spanning the hexagons of the pyrochlore lattice, is only generated at higher order. Significant second- and third-neighbour exchange can be present in real materials either intrinsically^19^,^20^, or via a partial cancellation of the leading terms^21^. One can show that for any SI state,


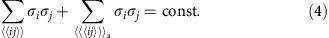


We thus see that two terms are not independent and when *J*_2_=*J*_3a_≡*J*′, they effectively cancel each other when in the SI manifold. Moving along the *J*_2_=*J*_3a_ line, the model moves away from the nearest-neighbour SI regime, but without lifting the degeneracy of the SI manifold. While SI persists as the ground state at low temperature for sufficiently small *J*′/*J*, eventually it gives way when another set of states crosses the SI manifold. This line of degeneracy also exists for analogous models with *N*-components spins, although how the termination manifests depends on the precise value of *N.* We thus refer to the model along this line as ESI. It will prove useful to write this model in terms of the charges *Q*_*I*_ as


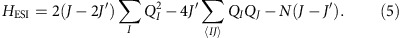


We see that *J*′>0 generates an attraction between nearest-neighbour charges of the same sign. This short-range attraction between charges will play a central role in understanding the ground and excited states of ESI .

**Figure 2:**
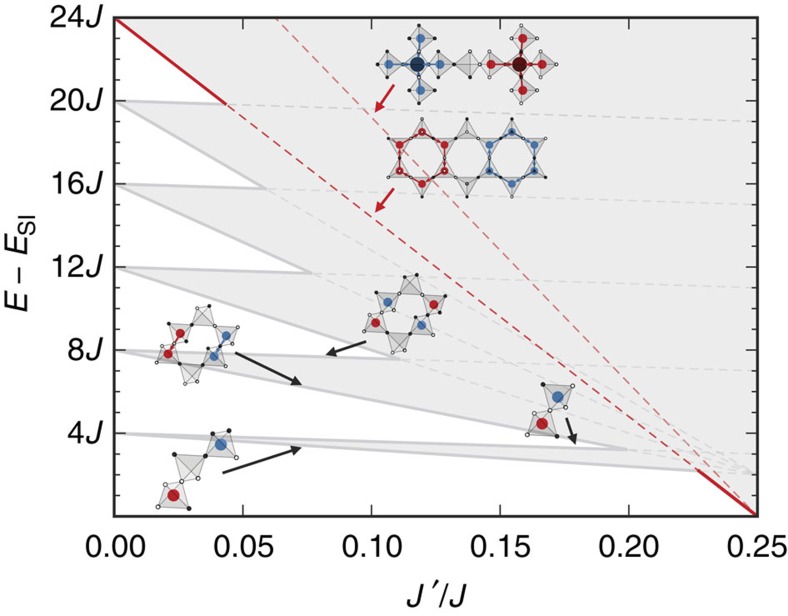
Collapse of excitations in extended spin ice. We sketch the structure of the excited states of the model of equation (3) along the *J*_2_=*J*_3a_≡*J*′ line. When *J*′ is non-zero, the highly degenerate bands of single- and double-charge states are split due to the nearest-neighbour attraction embodied in the second term in equation (5). For the low lying bands, we illustrate the charge arrangements that are favoured and those that are disfavoured by *J*′. Near the spin slush at *J*′/*J*∼1/4, an infinite set of excited states collapse to zero energy. We have illustrated two of the simplest examples, built from twelve charges, with energy shown by red lines.

One can show (see Supplementary Note 1) that the SI manifold persists until *J*′=*J*/4 for *J*′>0 and to *J*′=−*J*/2 for *J*′<0. Going along the *J*_2_=*J*_3a_ line towards negative values (away from the SS) in our model, the end point has a similar manifold of states (distinct from the SS states) to that studied in ref. 22. Specifically, at *J*′=−*J*/2, the ground states include all configurations with staggered charge *Q*_*I*_=*Q*_0_(−1)^*I*^. These are the ice states (*Q*_0_=0), the single-charge states (*Q*_0_=1)) and the all-in, all-out states (*Q*_0_=2). The collapse of excited states when approaching *J*′=*J*/4 is illustrated in Fig. 2. We show only the simplest examples that cross the ice manifold, but as we shall see, there are an infinite set of such states. We focus on the end point at *J*′=*J*/4 which we refer to as the SS model. At this special point, one can write the model (Eq. 4) as


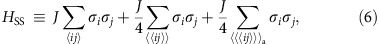



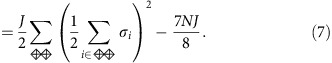


In this form, one notes a strong similarity to the SI model of equations (2), 4, except with the fundamental unit now being a pair of tetrahedra, indicated by , rather than a single tetrahedron.

### Ground-state manifold

The ground-state manifold of SS is most easily characterized in terms of the variables

Following equations (7), 10, any state with *P*_*i*_=±½ for all sites has the minimal energy −3*NJ*/4 and is in the ground-state manifold. Alternatively, we can write this in terms of the SI charges; associating each site *i* of the pyrochlore lattice with a nearest-neighbour bond 〈*IJ*〉 of the dual diamond lattice, one has *P*_*i*_=(−1)^*I*^(*Q*_*I*_−*Q*_*J*_)−*σ*_*i*_/2. From this expression for *P*_*i*_ in terms of the SI charges, it is clear that any SI state with *Q*_*I*_=0 for all sites also belongs to the SS manifold. In addition to the familiar SI states, many more states satisfy *P*_*i*_=±½. A naive enumeration of states for an isolated pair of tetrahedra shows that beyond the 18 ice states, there are an additional 52 states, 70 in total, that belong to the SS manifold. We caution that a Pauling-like estimate severely underestimates the degeneracy of the SS manifold. Given that the number of tetrahedron pairs is equal to the number of sites, one would estimate a residual entropy of *Nk*_B_(log 2+log(70/2^7^))∼0.0896*Nk*_B_. This reflects that the constraints provided by *P*_*i*_=±½ are much less independent than in SI where Pauling's estimate is accurate. These additional states include configurations with both single-charge (*Q*_*I*_=±1) and double-charge (*Q*_*I*_=±2) defects. The influence of the nearest-neighbour attraction of charges manifests here; pairs of like single charges can appear together, while double charges only appear with accompanying single charges of the same sign. One finds from equation (5) that the energy cost of having a charge can be compensated by the energy gain of having two neighbouring charges of the same sign.


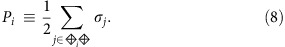


From these observations, we formulate rules for constructing states that satisfy *P*_*i*_=±½. We formulate these rules from the perspective of specifying non-ice tetrahedra (*Q*_*I*_≠0) states first, then populating the remaining tetrahedra with any compatible ice states afterward. The first rule for placing the non-ice, charged tetrahedra, is the single-charge rule: this states that the minority spin of a single charge, *Q*_*I*_=±1, must be connected to a tetrahedron carrying a single or double charge of the same sign. The second rule, the double-charge rule, states that a double charge *Q*_*I*_=±2 must have its four nearest-neighbour tetrahedra occupied by single charges of the same sign. Finally the neighbour rule requires that a single charge, *Q*_*I*_=±1, cannot have any single charges of opposite sign as nearest neighbours. Once single and double charges have been placed such that they satisfy the above three rules, one can assign the remaining tetrahedra any allowed ice rule, *Q*_*I*_=0, states. The first rule allows the single-charge tetrahedra to form branching tree-like structures where the minority spin of a given charge also belongs to the next charge in the structure. Each branch must terminate in some way compatible with the rules, so the minority spin must end up on another single charge. The possibilities for terminating a branch include looping back to itself, ending on a different branch or on one of the single charges attached to a double charge. Note that these single- and double-charge structures must exist for both signs of the charge to satisfy the global neutrality requirement ∑_*I*_ *Q*_*I*_=0. The third rule implies that charge structures of opposite sign must be separated by at least one ice rule obeying tetrahedron. An illustration of an SS state incorporating all of these features, restricted to a single [111] kagomé plane, is shown in Fig. 1.

### Thermodynamic and magnetic properties

**Figure 3:**
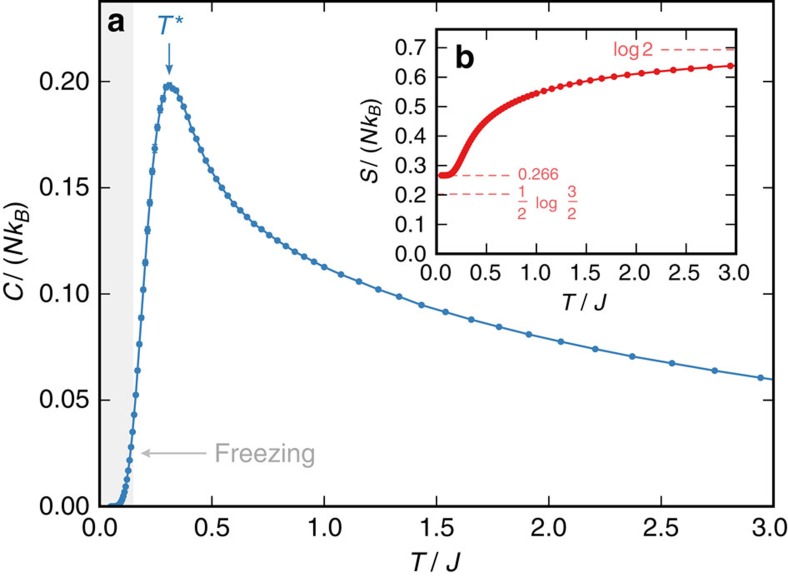
Specific heat and entropy of extended spin ice. Finite temperature (**a**) specific heat, *C*, and (**b**) entropy, *S*, of the spin slush model for a system of 10^3^ conventional cubic cells of the pyrochlore lattice. Entrance into the spin slush manifold is signalled by the peak in the specific heat at T*∼0.3*J*. Residual entropy as *T*→0 is *S*∼0.266*Nk*_B_. Freezing becomes apparent below *T*∼0.15*J*, as indicated by the shaded region.

With the ground states of SS identified, we now explore its finite temperature properties via classical Monte Carlo simulations using single-spin-flip dynamics, augmented with parallel tempering when appropriate. Basic thermodynamic quantities are shown in Fig. 3. The specific heat exhibits a broad peak at T*∼0.3*J*, reminiscent of the peak seen in SI. This peak signals the release of entropy as one begins to enter the SS ground-state manifold. This can be seen explicitly in the entropy in Fig. 3 where, below T*, the entropy approaches the constant value *S*_SS_∼0.266*Nk*_B_. As expected from the rules derived in the previous section, this is significantly higher than *S*_SI_∼0.202*Nk*_B_ found in SI. At these low temperatures severe freezing is encountered, preventing the simulations from reaching equilibrium below *T*∼0.15*J*. It is not obvious how to construct a non-local move that would sample the SS manifold efficiently. Including the SI loop move does aid equilibriation, but it is only effective in regions where no single- and double-charge defects are present, leaving the freezing problem for future work. The frozen states belong to the SS manifold and exhibit the single- and double-charge structures discussed in the previous section. We found no evidence of ordering in any of our simulations, be it conventional or via an order-by-disorder mechanism. Further, the specific heat and entropy are somewhat immune to this freezing problem, showing consistent behaviour between simulations. The magnetic properties are however more sensitive.

**Figure 4:**
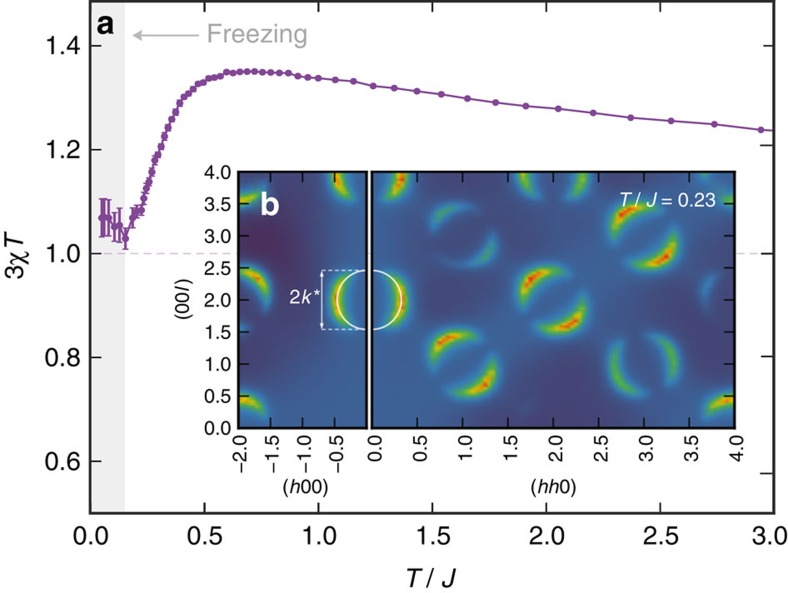
Magnetic properties of extended spin ice. (**a**) Finite temperature susceptibility χ of the spin slush model for a system of 10^3^ cubic cells. The susceptibility passes through maximum near *T*∼0.6*J* before settling into a Curie-like regime with 3*χT*∼1. Freezing becomes apparent below *T*∼0.15*J*, with the susceptibility depending on the detailed spin configuration of the frozen state. (**b**) Transverse moment–moment correlation function *I*(**k**) defined in equation (9), for the spin slush model at *T*=0.23*J* for a system of 24^3^ cubic cells. Cuts in the [*hhl*] and [*h*0*l*] planes are shown. Correlations are peaked on spherical surfaces of radius k*∼0.5(2*π*/*a*) where *a* is the size of a cubic unit cell. These spheres are centred on the locations of the pinch-points in spin ice.

The simplest probe of the magnetic behaviour is the uniform susceptibility, χ, shown in Fig. 4, for the moments 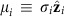 pointing in/out of the tetrahedra along the local [111] direction 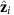. At both low and high temperatures, one finds Curie-like behaviour, with 3*χT* constant, separated by a broad peak at *T*∼*O*(*J*). The constant approached as *T*→0 depends on the details of how the system freezes. This varies between simulations, taking on a distribution of values clustered around 3*χT∼1*, reflected in the large error bars in Fig. 4. A more detailed probe of the magnetic structure are the spin–spin correlations, as can be investigated via neutron scattering. Recall that in SI the appearance of sharp pinch-points^23^ in the transverse moment–moment correlation function


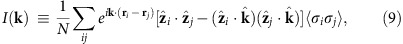


signals the development of long-range dipolar spin–spin correlations. In SS, one finds sharp features in *I*(**k**) distinct from such pinch points. As shown in Fig. 4, below T* *I*(**k**) develops into sharp rings centred on zone centres in a given plane of reciprocal space. In the full [*hkl*] space, these features lie approximately on spheres, reminiscent of an isotropic liquid. As discussed in Supplementary Note 2, this analogy is even more striking in the structure factor of the SI charges *Q*_*I*_ where the intensity is approximately uniform across the sphere, as shown in Supplementary Figs 2 and 3. The wave-vector 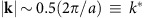, where *a* is the size of the conventional cubic unit cell, indicates these correlations have a characteristic length of two cubic cells and thus represent intermediate scale correlations. These correlations are consistent with the typical size of the charged structures that appear in the ESI manifold. Indeed, as seen in Fig. 1, even the smallest of these structures can span several cubic unit cells.

These simulations confirm that the SS model does not order and the SS manifold shows all the rich charge structures at intermediate length scales implied by the SS rules. Indeed, at low temperatures, a significant fraction of tetrahedra, ∼30–35%, carry single charges, while a smaller but finite fraction, a per cent or so, carry double charges. Similar to the susceptibility, the amount of single and double charges present at low temperatures varies somewhat from run to run, a consequence of the severe freezing problem. To better understand this issue, we now look more closely at the low-temperature dynamics of SS.

### Dynamics and spin slush

To reflect the physics of real systems with local dynamics, we employ only single-spin flip, Metropolis dynamics, although we expect any local dynamics to give qualitatively the same behaviour. We primarily consider the site-resolved auto-correlation functions, defining *A*_*i*_(*t*)≡〈*σ*_*i*_(*t*_0_)*σ*_*i*_(*t*_0_+*t*)〉, where *σ*_*i*_(*t*) is the Ising spin at a given Monte Carlo sweep *t* at site *i*, averaging over many initial times *t*_0_. Generically, one would expect exponential relaxation 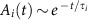 with a characteristic relaxation time *τ*_*i*_. Indeed this is found in SI, with the relaxation time being site-independent, with *τ*_*i*_∼*τ* and increasing exponentially as temperature is lowered^16^.

**Figure 5:**
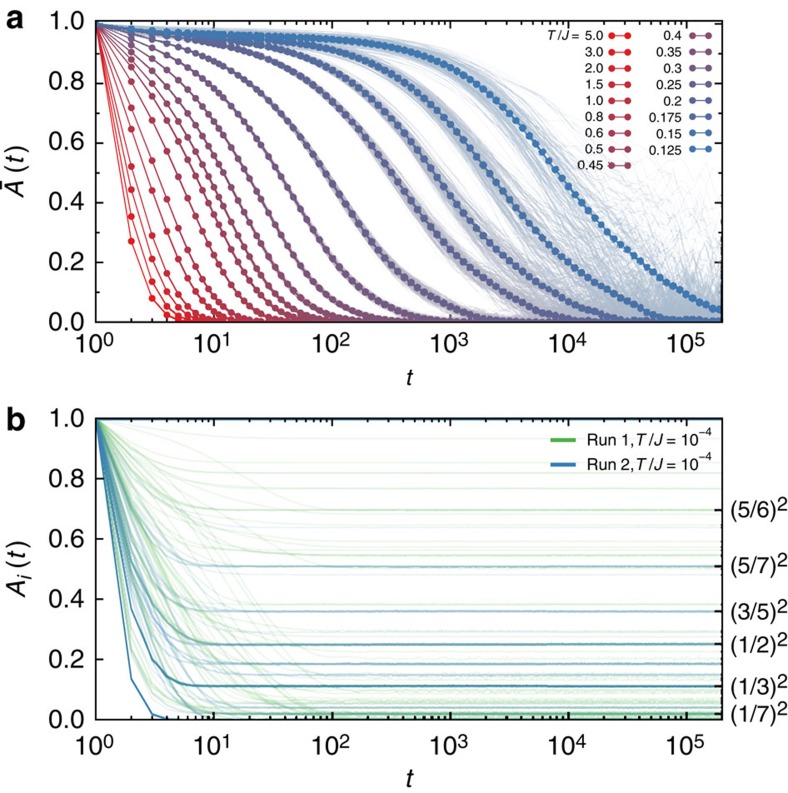
Auto-correlation functions in extended spin ice. (**a**) Site-averaged auto-correlation function 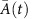 at various temperatures for a system of 8^3^ cubic cells. As we approach low temperatures, the relaxation time grows exponentially. Short-time dynamics is apparent in the initial decrease of 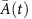 for *t*≲10^2^. The thin curves show a sample of the individual site-resolved *A*_*i*_(*t*) at each temperature, showing increasing levels of heterogeneity for *T*≲T*. (**b**) Site-resolved auto-correlation functions *A*_*i*_(*t*) at the very low temperature *T*=10^−4^*J*. We show two distinct annealed runs of a system of 8^3^ cubic cells. Aside from essentially frozen spins with *A*_*i*_(*t*)=1, one finds many spins that relax over time scales of 10^1^ or 10^2^ sweeps. At long times the auto-correlation functions reach constant values *A*_*i*_(∞) that are clustered about the squares of simple, rational numbers (see text).

In contrast to SI, the dynamics in SS vary strongly from site to site. As temperature is lowered, most of the sites freeze, with their relaxation times becoming very long, similar to what is found in SI^15^,^16^. This can be seen in the site-averaged auto-correlation function 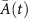 shown in Fig. 5. However, there are clear differences, namely in the initial decrease and plateau in 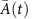 at short times as well as in the larger site to site variance in *A*_*i*_(*t*) at low temperatures. We can understand this behaviour by looking at the *T*→0 limit; one finds that a fraction of sites remain highly dynamic down to very low temperatures. This is illustrated in Fig. 5, where the site-resolved auto-correlation functions are shown for *T*=10^−4^*J*. The frozen spins have *A*_*i*_(*t*)=1 at all times, while the unfrozen spins have *A*_*i*_(*t*) relaxing in 10^1^–10^2^ Monte Carlo sweeps to a constant value lim_*t*→∞_*A*_*i*_(*t*)≡*A*_*i*_(∞)<1. To be precise, for the long-time limit lim_*t*→∞_*A*_*i*_(*t*), we mean *t*⩾1 but still much smaller than the slow timescale ∼*O*(*e*^*J*/*T*^). A non-zero value of *A*_*i*_(∞)<1 indicates that, while fluctuating, on average more time is spent in one of the states *σ*_*i*_=±1 than the other. For example, if *σ*_*i*_ is sampling uniformly from values *σ*^(1)^,…, *σ*^(*m*)^ as a function of time, then 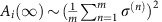 at long times. Figure 5 shows that the long-time values *A*_*i*_(∞) cluster about the squares of rational numbers, as would be expected from the above discussion. In these annealed simulations, the frozen spins make up the bulk of the system, while the number of unfrozen, dynamic spins is on the order of a few per cent.

**Figure 6:**
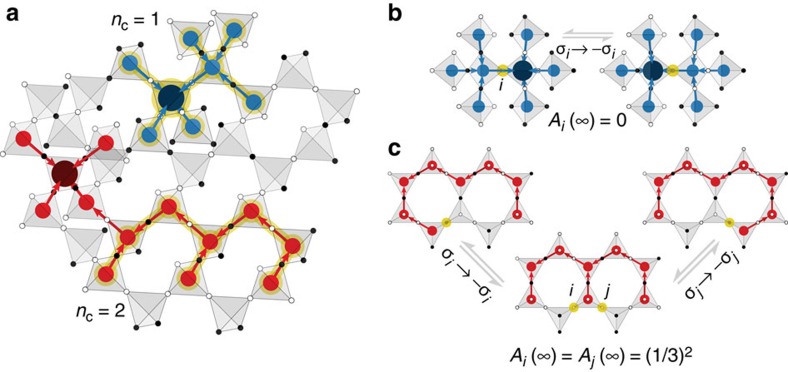
Dynamical clusters in spin slush. We illustrate some of the dynamical clusters that can appear in the spin slush ground-state manifold. In (**a**) we show an example of part of a state with two such clusters, one containing a single dynamical spin (*n*_c_=1) and the other containing two dynamical spins (*n*_c_=2), with the dynamical spins and the surrounding charges highlighted in gold. In (**b**) we show the accessible states of an *n*_c_=1 dynamical cluster where the two states yield an average spin of zero and thus *A*_*i*_(∞)=0. In (**c**) we show the *n*_c_=2 case, where one finds three accessible states with an average spin of ±1/3 and thus *A*_*i*_(∞)=*A*_*j*_(∞)=(1/3)^2^. In (**b**,**c**) the flippable spins for each state are highlighted in gold.

To better understand these dynamic spins, we examine their real space structure. We find that these spins are spatially correlated, forming clusters of varying size *n*_c_. A dynamical cluster is defined by a set of spins, where lim_*t*→∞_*A*_*i*_(*t*)<1 and each spin is connected by a first, second or third neighbour bond to another spin in the cluster. The SS state at low temperature is thus a mixture where regions of frozen and unfrozen spins coexist. Dynamical clusters built from a small number of sites can be identified directly from the SS rules. Figure 6 shows an SS ground state containing several of these dynamical clusters. For example, one has a single site that can be flipped while preserving all of the SS rules, representing an *n*_c_=1 dynamical cluster. A larger *n*_c_=2 cluster is also shown, where two spins can be flipped, though not independently. For both these examples we note that a large number of surrounding frozen spins are needed to construct these dynamical clusters. A naive counting for the *n*_c_=1 case yields a fraction of unfrozen to frozen spins of ∼1/25∼4%, comparable to the few per cent average of unfrozen spins observed in our annealed simulations. These examples represent only a small subset of the possible dynamical clusters that can be constructed in the SS manifold. As described in Supplementary Note 3, there are several ways to construct dynamical clusters of arbitrary size (see Supplementary Fig. 4) as well as illustrations of the time evolution of dynamical clusters in simulations of small systems. The presence of such dynamical clusters is not specific to the single-spin-flip dynamics used; for example, analogous dynamical clusters can be constructed for spin-exchange dynamics, as illustrated in Supplementary Fig. 5, and we expect the same holds true for any local dynamics.

## Discussion

Outside of any pure theoretical interest, one may be concerned with the fine-tuning required to reach the SS phase. As in SI^13^, though the precise point in phase space may be difficult to reach in a material realization, the nearby regions in phase space may be controlled primarily by the SS physics. Understanding the SS manifold then allows one to understand the surrounding phases and their higher temperature properties as perturbations to the SS model. Here we discuss two types of such perturbations: deviations from the *J*_2_=*J*_3a_ ESI line and quantum terms, such as transverse field or exchange.

While the effects of finite second- and third-neighbour exchange on similar models has been studied extensively^24^,^25^,^26^,^27^,^28^, the regime along the ESI line and near the SS point remains largely unexplored. We find four neighbouring phases; the simplest are a (½, ½, ½) ordered phase expected from the *J*_3a_→+∞ limit that appears for *J*_3a_>*J*/4 and a ferromagnetic SI state expected from the *J*_3a_→−∞ limit that appears for *J*_3a_<*J*/4. For *J*_2_<*J*/4 one finds a set of layered states (These are related to, but not identical to the layered states discussed for the *J*-*J*_2_ classical Heisenberg model of ref. 26). with sub-extensive degeneracy ∼2^*L*^. For *J*_2_>*J*/4, one finds a complex incommensurate ordering with wave-vector along [*h*00] or equivalents. The SS manifold ties these phases together, all of which are drawn from the SS ground-state manifold, with *P*_*i*_=±½ for all pairs of tetrahedra, and extend over large regions of parameter space. We leave the detailed investigation of these neighbouring phases and other perturbations (such as *J*_3b_, dipolar interactions and so on) for future studies.

The effect of quantum non-Ising interactions on SS is potentially much richer than in SI. In the latter, the addition of transverse exchange or transverse field induces tunnelling within the SI manifold yielding a *U*(1) quantum spin liquid^29^,^30^,^31^,^32^. This quantum spin liquid is described by an emergent electrodynamics, complete with a gapless photon excitation^29^. However, the associated energy scale of the quantum spin liquid is very small, due to tunnelling only appearing at high order in perturbation theory, confining its effects to very low temperatures and close proximity to the SI point^30^,^33^. In the SS, quantum dynamics appear at first order in perturbation theory (see Supplementary Note 4), and thus we expect them to be more significant than in SI. The presence of these first-order matrix elements is a direct reflection of the single-spin-flip and spin-exchange dynamics of the SS manifold. Even with such mixing, when the perturbed Hamiltonian is projected into the SS manifold it still breaks up into infinitely many disconnected blocks, representing sets of states reachable by such local moves. The simplest blocks correspond to a small number of dynamical clusters well-separated by frozen regions. For example, there can be many *n*_c_=1 clusters as in Fig. 6, each with two states, corresponding to the freely flippable spin 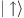 and 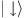 for each cluster. Application of a transverse field 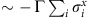 mixes the two states and gives a ground state of 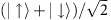 with energy gain of −Γ per dynamical spin. Other blocks correspond to more complicated dynamical clusters with more spatially extended structures. For example, for the large linear clusters discussed in Supplementary Note 3, the energy gain per dynamical spin is smaller, approaching approximately −2Γ/*n*_c_ for clusters of size *n*_c_ (see Supplementary Note 4 and Supplementary Fig. 6 for an explicit example of this). More exotically, one can even construct states where a single dynamical cluster of size *n*_c_∼*O*(*N*) encompasses nearly all of the spins in the system. Examples of such larger and more complex dynamical clusters are illustrated in Supplementary Movies 1 and 2. Similar considerations apply for transverse exchange 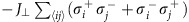. A key difference is that odd-sized dynamical clusters are guaranteed to have degenerate ground states due to Kramers' theorem. In the exchange case, the *n*_c_=1 clusters thus contain free spins and gain no energy.

We thus conclude that for quantum SS, the ground states favoured at first order in perturbation theory will depend on the ground-state energies of this zoo of clusters as well as their effective packing fractions. We leave the detailed resolution of these non-trivial questions to future work. As this model is free of the sign problem, some of these questions should be addressable through quantum Monte Carlo simulations for both a ferromagnetic transverse exchange (*J*_±_>0) or an arbitrary transverse field. The physics of the above dynamical clusters and the heterogeneous freezing could potentially enlighten our understanding of the phenomena of persistent dynamics in highly frustrated magnets^34^. In a more concrete setting, one may speculate that the SS could be connected to the physics observed in the quantum spin liquid candidate Tb_2_Ti_2_O_7_. A tantalizing clue are the short-range correlations^35^ at wave-vector (½, ½, ½) seen in Tb_2_Ti_2_O_7_ and the (½, ½, ½) phase obtained by perturbing SS .

In summary, we have identified SS, a cooperative paramagnet on the pyrochlore lattice found by extending SI with further neighbour exchanges. This classical Ising model serves as a simple example of freezing and dynamical heterogeneity in a clean, disorder-free system. The features present in the magnetic correlations and the unusual low-temperature dynamics could prove useful in understanding such physics in real materials. We note that during the review of this article^36^, which also studies the ESI model and the SS point, appeared.

## Methods

### Monte Carlo simulations

For all Monte Carlo simulations, we used the standard Metropolis updating scheme with single-spin flip moves. For thermodynamic quantities, we simulated systems of *N*=16*L*^3^ spins in *L*^3^ conventional cubic unit cells of the pyrochlore lattice under periodic boundary conditions with linear size up to *L*=10. Typically, we used *O*(10^6^) sweeps to anneal the system to each temperature and thermalize, then an additional *O*(10^6^) sweeps were used to compute observables. Error estimates were computed via the bootstrap method. For spin–spin and charge–charge correlation functions, we simulated larger systems of size up to *L*=24, but only *O*(10^5^) sweeps were needed to obtain accurate results. In both cases, we also used parallel tempering moves after each sweep to aid equilibriation. Longer simulations on smaller system sizes, with *O*(10^7^) to *O*(10^8^) sweeps produce results consistent with the shorter simulations on the larger systems. For dynamical quantities, a comparable number of sweeps and system sizes were used, except without the use of parallel tempering. To access the very low-temperature auto-correlation function, we first slowly annealed the system to *T*/*J*=10^−4^, guaranteeing that an SS ground state was reached, then followed the same protocol as the higher temperature simulations. This was repeated many times; two of these simulations are described in the main text.

### Data availability

Raw data for any of the results reported in the text are available from the authors upon request.

## Additional information

**How to cite this article:** Rau, J. G. & Gingras, M. J. P. Spin slush in an extended spin ice model. *Nat. Commun.* 7:12234 doi: 10.1038/ncomms12234 (2016).

## Supplementary Material

Supplementary Information Supplementary Figures 1-6 and Supplementary Notes 1-4

Supplementary Movie 1 Illustration of dynamical spins in simulations of small systems (128 spins, 2^3^ cubic cells). We show the time evolution of a state of the spin slush manifold obtained through annealing to very low temperature. At a given time the unfrozen, flippable spins are highlighted in gold. At each Monte Carlo time step we flip one of these unfrozen spins at random, omitting time steps where a flip of a frozen spin was rejected. In Supplemental Movie 1, we show a highly dynamical state consisting of only single charge structures. Over the time evolution, the active, dynamical spins are distributed over many different sites of the system.

Supplementary Movie 2 Illustration of dynamical spins in simulations of small systems (128 spins, 2^3^ cubic cells). We show the time evolution of a state of the spin slush manifold obtained through annealing to very low temperature. At a given time the unfrozen, flippable spins are highlighted in gold. At each Monte Carlo time step we flip one of these unfrozen spins at random, omitting time steps where a flip of a frozen spin was rejected. In Supplemental Movie 2, we show state that contains dynamical clusters of single charges and contains a simple n_c_=1 double charge structure. As time evolves, the dynamics of the double-charge is activated and deactivated as the surrounding single charges fluctuate about their positions.
