# Supplementary material for: Spin slush in an extended spin ice model
Source: Nat Commun. 2016 Jul 29;7:12234. doi: 10.1038/ncomms12234 (PMC4974571; doi:10.1038/ncomms12234)
Supplement: Supplementary Information — Supplementary Figures 1-6 and Supplementary Notes 1-4 [file ncomms12234-s1.pdf]

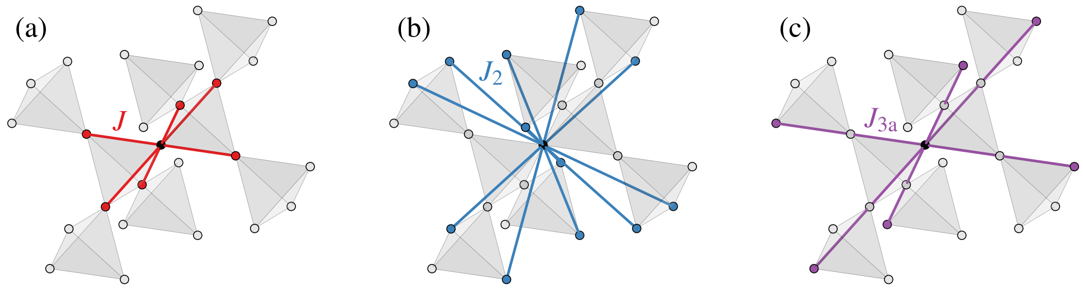

Supplementary Fig. 1. **First, second and third nearest neighbours on the pyrochlore lattice:** We show the bonds corresponding to (a) the first nearest neighbours (J), (b) the second nearest neighbours (J_2) and (c) the colinear third nearest neighbours (J_{3a}).

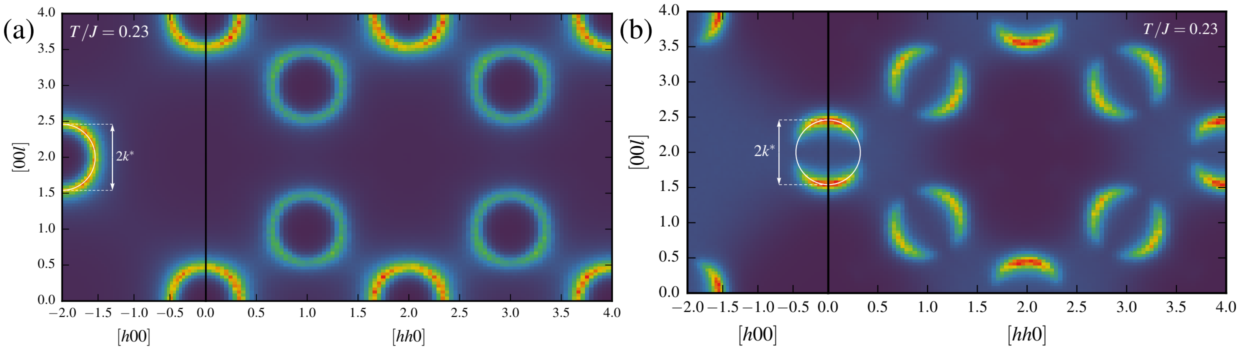

Supplementary Fig. 2. **Spin and charge correlation functions in spin slush:** (a) Here we show correlation function of the spin ice charges in the spin slush state for a system of 24^3 cubic unit cells. Liquid-like correlations exist with characteristic wave-vector k^*. In contrast with the spin-spin correlation function, the intensity of the sharp features are much more uniform. (b) Here we show the spin-spin correlation function as defined in Supplementary Eq. (4) for a system of 24^3 cubic unit cells. Liquid-like correlations exist with characteristic wave-vector k^*.

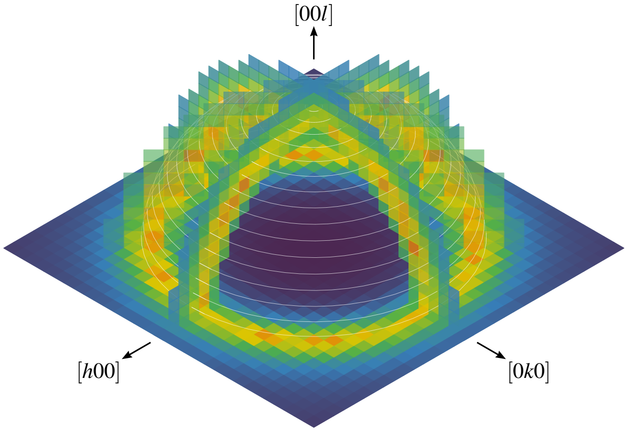

Supplementary Fig. 3. **Liquid-like charge correlations in spin slush:** We illustrate the full structure of the charge-charge correlations function $S_Q(\mathbf{k})$, defined in Supplementary Eq. (3), at $T/J = 0.23$ for a system of 24^3 cubic cells. The high intensity regions approximately follow a sphere of radius $k^* \sim 0.5(2\pi/a)$ (shown by white lines) centered about zero wave-vector, reminiscent of an isotropic liquid.

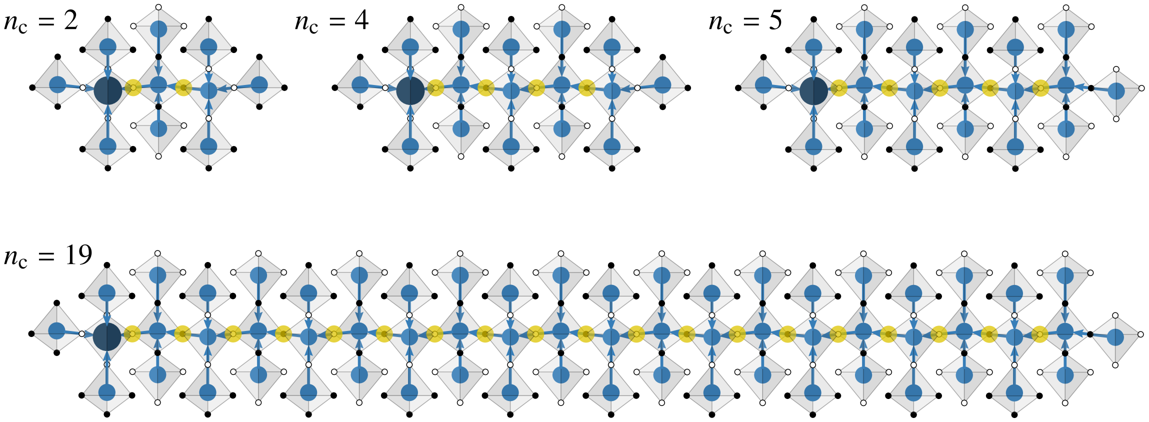

Supplementary Fig. 4. **Large linear single-spin flip dynamical clusters:** We illustrate generalizations of the $n_c = 1$ dynamical cluster discussed in the main text to arbitrary size n_c. By flipping neighbouring spins, the double-charge can hop along the length of these chains, rendering all the spins highlighted in gold dynamical.

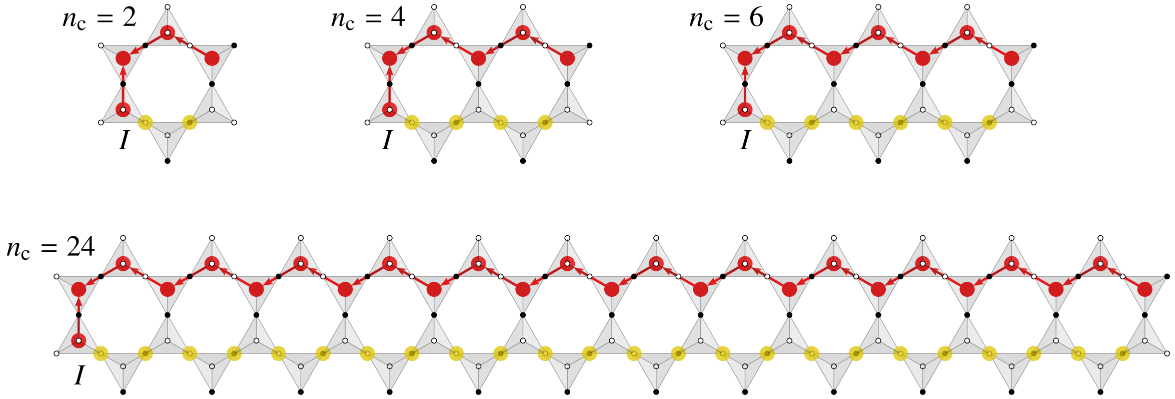

Supplementary Fig. 5. **Large linear spin-exchange dynamical clusters:** We illustrate a family of dynamical clusters for spin-exchange dynamics with arbitrary size n_c. By swapping pairs of neighbouring spins, the single-charge at tetrahedron I attached to the chain can hop along the length of the chain, rendering all the spins highlighted in gold dynamical.

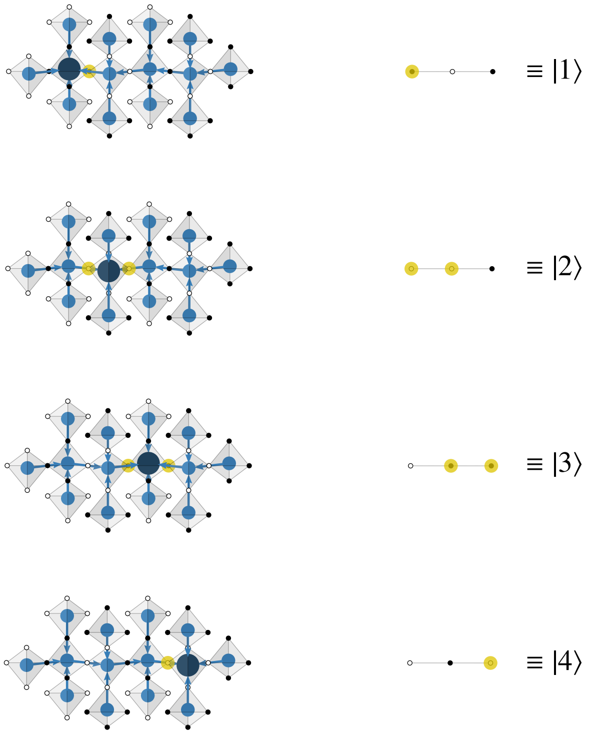

Supplementary Fig. 6. **States accessible by application in a transverse field:** We show the four states of an $n_c = 3$ cluster that are linked by the application of a transverse field as shown in Supplementary Eq. (5). The full cluster in shown on the left, while the three relevant dynamical spins are shown on the right. The spins highlighted in gold in a given state are flippable.

SUPPLEMENTARY NOTES

Supplementary Note 1

Ground states along extended spin ice line

In this section we sketch a proof that the ground states for $0 \leq J' < J/4$ are the two-in/two-out spin ice manifold. First recall the definition of J, J_2 and J_{3a}, as explicitly shown in Supplementary Fig. 1. Consider the decomposition of H into contributions local to each pair of tetrahedra, i.e. one writes the total energy as $H \equiv \sum_{\langle IJ\rangle} \mathscr{H}_{IJ}(\sigma)$ where

$$\mathscr{H}_{IJ}(\sigma) = \frac{J}{4}\left[\sum_{\langle ij\rangle\in I} \sigma_i\sigma_j + \sum_{\langle ij\rangle\in J} \sigma_i\sigma_j\right] + J_2 \sum_{\langle\langle ij\rangle\rangle\in\langle IJ\rangle} \sigma_i\sigma_j + J_{3a} \sum_{\langle\langle\langle ij\rangle\rangle\rangle\in\langle IJ\rangle} \sigma_i\sigma_j. \quad (1)$$

Note that $\mathscr{H}_{IJ}(\sigma)$ depends only on the seven spins that belong to the tetrahedron pair $\langle IJ\rangle$. For a given choice of J, J_2 and J_{3a} we define the ground state energy of this seven site system as E_0^*. Now suppose we have some configuration of spins σ_i^* that has the following property: each pair of tetrahedra has minimum energy with respect to $\mathscr{H}_{IJ}(\sigma)$, that is $\mathscr{H}_{IJ}(\sigma^*) = E_0^*$ for all pairs $\langle IJ\rangle$. Informally, this states that the two tetrahedron ground states can be tiled across the full lattice in a consistent way. We then can infer that σ^* is a ground state of the full model H. The argument is a simple inequality; since $\mathscr{H}_{IJ}(\sigma) \geq E_0^*$ for *all* states σ by definition, we can say that

$$H = \sum_{\langle IJ\rangle} \mathscr{H}_{IJ}(\sigma) \geq \sum_{\langle IJ\rangle} E_0^* = E^*, \quad (2)$$

where E^* is the energy of the state σ^*. Thus $H \geq E^*$ for all states σ and so σ^* is part of the ground state manifold. Enumerating the 2^7 states of the two tetrahedron system and minimizing $\mathscr{H}_{IJ}(\sigma)$, one can thus show that the $Q_I = 0$, SI states are the ground states for $0 \leq J' < J/4$.

Supplementary Note 2

Spin and charge correlations in spin slush

Here we provide further evidence of the intermediate range correlations in the SS phase at $J' = J/4$ shown in Fig. 4 of the main text. In Supplementary Fig. 2 we show the charge-charge correlation function

$$S_Q(\mathbf{k}) \equiv \frac{1}{N_d} \sum_{IJ} e^{i\mathbf{k}\cdot(\mathbf{R}_I-\mathbf{R}_J)} \langle Q_I Q_J\rangle, \quad (3)$$

where $N_d = N/2$ is the number of dual diamond lattice sites and $\mathbf{R}_I$ are the sites of the dual diamond lattice. The sharp, liquid-like features found in $I(\mathbf{k})$ are present, but with a more uniform intensity, consistent with the charges forming intermediate range structures due to the short-range

attraction induces by J_2 and J_{3a}. A three-dimensional view, in the full [hkl] space is shown in Supplementary Fig. 3. In Supplementary Fig. 2 we show the spin-spin correlation function, without the complicating effects of the transverse projection or local axes for the moments. Explicitly, we compute

$$S(\mathbf{k}) \equiv \frac{1}{N} \sum_{ij} \langle \sigma_i \sigma_j \rangle e^{i\mathbf{k}\cdot(\mathbf{r}_i - \mathbf{r}_j)}. \tag{4}$$

The same sharp, ring-like features are seen in each scattering plane, as in the transverse moment-moment correlator shown in Fig. 4 of the main text.

Supplementary Note 3

Zoo of dynamical clusters

We illustrate some large dynamical clusters for single-spin flip dynamics that can be constructed directly from the SS rules. First, we consider a generalization of the $n_c = 1$ cluster shown in Fig. 6 of the main text. One can easily append further single charges to the cluster to enlarge it. We show several of these larger linear clusters in Supplementary Fig. 4. For each, all of the central spins (highlighted in gold) are dynamic, though not simultaneously. At any given point in time, only the central spins that belong to the double charge tetrahedra can be flipped. This allows the double charge to effectively hop randomly along the chain. We note that the topology of these clusters is flexible; the linear geometry shown here is for illustration purposes, it can be bent at any of the single charges. More complicated structures of this sort can be setup (trees, loops, etc) that allow the double charge to hop. Similar clusters can be constructed for spin-exchange dynamics, where two neighbouring spins are swapped. We illustrate one of these large clusters in Supplementary Fig. 5. The protruding single-charge at tetrahedron I on the far left can hop along the chain by swapping neighbouring pairs of spins.

Supplementary Note 4

Effect of transverse field

To gain some understanding of the quantum effects acting within the SS manifold, we consider a simple, non-trivial cluster under the application of a transverse field at first order in perturbation theory. Similar considerations apply for transverse exchange. We start with the interaction:

$$V \equiv -\Gamma \sum_i \sigma_i^x, \tag{5}$$

with $\Gamma > 0$. At first order we must diagonalize the interaction V projected into the SS manifold. The connection to the single-spin flip dynamics is manifest here; the non-zero matrix elements at

first-order in perturbation theory correspond to those spins that can be flipped while remaining in the ground state manifold, i.e. the dynamical spins. We will ignore the rest of the lattice in this analysis, treating only this one block of the projected interaction V describing a single dynamical cluster. We take the cluster shown in Supplementary Fig. 6 where there are $n_c = 3$ dynamical spins and four accessible states. We define a projection into these four states as

$$P \equiv \sum_{m=1}^{4} |m\rangle \langle m| . \tag{6}$$

By inspection (see Supplementary Fig. 6), the matrix elements within this subspace are easily found to be

$$\begin{aligned} PV|1\rangle &= -\Gamma|2\rangle, & PV|2\rangle &= -\Gamma(|1\rangle + |3\rangle), \\ PV|3\rangle &= -\Gamma(|2\rangle + |4\rangle), & PV|4\rangle &= -\Gamma|3\rangle. \end{aligned} \tag{7}$$

More explicitly, the first-order correction to the energy in the cluster is obtained from diagonalizing the matrix

$$PVP \equiv -\Gamma \begin{pmatrix} 0 & 1 & 0 & 0 \\ 1 & 0 & 1 & 0 \\ 0 & 1 & 0 & 1 \\ 0 & 0 & 1 & 0 \end{pmatrix}. \tag{8}$$

This can be done easily, with energy corrections

$$\delta E = \pm\Gamma\left(\frac{1-\sqrt{5}}{2}\right), \quad \pm\Gamma\left(\frac{1+\sqrt{5}}{2}\right). \tag{9}$$

The lowest energy configuration of this cluster corresponds to $\delta E = -\Gamma(1+\sqrt{5})/2$ with eigenvector

$$|0\rangle \propto 2(|1\rangle + |4\rangle) + (1+\sqrt{5})(|2\rangle + |3\rangle). \tag{10}$$

More generally, for such a linear cluster of n_c spins, this is equivalent to a hopping problem on $n_c + 1$ sites with open boundary conditions. Since the non-zero off-diagonal elements are constant, the eigenvalues and eigenvectors can be found recursively. The energies are given by

$$\delta E_k = -2\Gamma \cos\left(\frac{\pi k}{n_c + 2}\right), \tag{11}$$

for $k = 1, 2, \ldots, n_c + 1$. The ground state corresponds to $k = 1$ and takes the form

$$|0\rangle \propto \sum_{m=1}^{n_c+1} \sin\left[\frac{m\pi}{n_c + 2}\right] |m\rangle . \tag{12}$$

This state is delocalized across all the $n_c + 1$ states, with more weight in the states away from the end states $|1\rangle$ and $|n_c + 1\rangle$. At large $n_c \gg 1$ the energy gain due to the transverse field is thus

$$\delta E_1 = -2\Gamma \cos\left(\frac{\pi}{n_c + 2}\right) \approx -2\Gamma + O(n_c^{-2}), \tag{13}$$

or $\sim -2\Gamma/n_c$ per dynamical site, as quoted in the main text. Periodic versions of these dynamical clusters can also be constructed by connecting the ends and can be analyzed similarly.
